# The Expression of Pax6 Variants Is Subject to Posttranscriptional Regulation in the Developing Mouse Eyelid

**DOI:** 10.1371/journal.pone.0053919

**Published:** 2013-01-10

**Authors:** Fangyu Shi, Yannan Fan, Laiguang Zhang, Lu Meng, Huifang Zhi, Hongyu Hu, Aixin Lin

**Affiliations:** State Key Laboratory for Agrobiotechnology, College of Biological Sciences, China Agricultural University, Beijing, China; Instituto Gulbenkian de Ciência, Portugal

## Abstract

Pax6 is a pivotal transcription factor that plays a role during early eye morphogenesis, but its expression and function in eyelid development remain unknown. In this study, the expression patterns of Pax6 mRNA and protein were examined in the developing mouse eyelid at embryonic days 14.5, 15.5, and 16.5. The function of Pax6 in eyelid development was determined by comparing it to that in the eyes-open-at-birth mutant mouse. In the normally developing eyelid, Pax6 and Pax6(5a) mRNA levels were low at E14.5, increased at E15.5, and then declined at E16.5, accompanied by a change in the Pax6/Pax6(5a) ratio. Pax6 protein was mainly located in the mesenchyme and conjunctiva. It was expressed at low levels in the epidermis at E14.5, severely reduced at E15.5, but re-expressed in the keratinocyte cells of the periderm at E16.5. In contrast, Pax6 and the Pax6/Pax6(5a) ratio were considerably higher with strong nuclear expression in the mutant at E15.5. Next, we examined the relationship of Pax6 to epidermal cell proliferation, migration, and the associated signalling pathways. The Pax6 protein in the developing eyelid was negatively correlated with epidermal cell proliferation but not migration, and it is in contrast to the activation of the EGFR-ERK pathway. Our in vivo data suggest that Pax6 expression and the Pax6/Pax6(5a) ratio are at relatively low levels in the eyelid, and acting as a transcription factor, Pax6 is required for the initiation of eyelid formation and for differential development of the keratinised cells in the closed eyelid. The Pax6 protein is likely to be controlled by the EGFR-ERK pathways. An abnormal increase in Pax6 expression and the Pax6/Pax6(5a) ratio due to alteration of the pathway activity could suppress epidermal cell proliferation leading to the eyes-open-at-birth defect. This study offers insight into the function of the Pax6 protein in eyelid development.

## Introduction

Pax6 is a pivotal transcription factor for vertebrate eye development [1], [2], [3]. In the early mouse embryo, Pax6 mRNA is expressed in a large part of the head surface ectoderm at embryonic day 8.0 (E8.0) and is subsequently restricted to the lens placode, nasal placode, and adjacent tissue at E9.5 [4]. Thereafter, Pax6 mRNA is continually expressed in some parts of the eye that are derived from the surface ectoderm (containing the lens and cornea), and it is also in the retina and iris at E15.5 [4], [5].

The function of Pax6 is dose-dependent in the mouse, and the Pax6 gene dosage has been shown to affect the differentiation and maintenance of ocular tissues [6], [7]. A homozygous mutation of Pax6 in the Sey mouse leads to a deficiency in eye and nose formation, and the mouse dies rapidly after birth [8]. The reduction of Pax6 levels in the heterozygous mouse causes delayed lens placode formation and a smaller lens [7]. Interestingly, Pax6 overexpression also influences eye development [9], [10]. PAX77, a transgenic mouse line with several copies of the human Pax6 locus, displays defects in its cornea, retina, iris, and ciliary body [11].

The eyelid, which is derived from the head surface ectoderm [12], is comprised of the epidermis (the outer surface of the eyelid), conjunctiva (the inner surface of the eyelid) and periderm (overlying the epidermis). At E11.5, the eyelid begins to protrude; the eyelid folds cover a part of the cornea and grow towards each other from E14 to E15 [13]. After 1 day, the upper and lower eyelids cover over the cornea and fuse together [14]. Defects in eyelid formation or closure lead to an obvious eyes-open-at-birth (EOB) phenotype in mice, which is caused by the alteration of two signalling pathways, TGFα/EGFR-ERK and TGF-β/ActivinβB [15], [16], [17].

To date, no data are available to show the expression pattern and function of Pax6 in the developing mouse eyelid. In this study, we investigate Pax6 expression and its pattern of regulation, as well as its function in the eyelid of wild-type mouse in parallel with a new EOB mutant mouse (EOB-5) during eyelid development. In addition, we analyse the linkage of Pax6 with signalling pathways using a careful comparison of the protein expression levels.

## Materials and Methods

### Mice and Embryos

C57BL/6 mice were purchased from the Animal Center of the Institute of Genetics and Developmental Biology (Chinese Academy of Sciences). The EOB-5 mutant mice were derived from an inbred transgenic line in our lab with a mixed background of C57BL/6 and DBA/2, which exhibit an eyes-open-at-birth phenotype. The EOB-5 mutant mice are the third backcross generation with C57BL/6. The mutation is recessive, which was confirmed by mating experiments, and it is closely linked with our foreign gene (green fluorescent protein driven by the oct4 promoter), which is located on chromosome 5 (data unpublished). Noon of the day when the vaginal plug was detected was considered as embryonic day (E) 0.5 of development. All animal studies were approved by the animal welfare committee of China Agricultural University with the approval number SK077.

### Immunohistochemistry and Immunofluorescence

The heads of E14.5, E15.5, and E16.5 embryos were fixed in 4% paraformaldehyde for 3 hours at room temperature, dehydrated, embedded in paraffin and cut into 5 µm sections. For immunohistochemistry, the sections were incubated with primary antibodies at 4°C overnight followed by an HRP-conjugated secondary antibody. For immunofluorescence, the sections were incubated with a FITC-conjugated secondary antibody (CW0114). The primary antibodies were PAX6 (centre) (AP6929c, Abgent Inc.), p-EGFR (BS4064, Bioworlde), p-ERK (BS4621, Bioworlde), and CTCF (5707-1, Epitomics, Inc.).

### Cell Proliferation Assays

The sections were incubated with an anti-PCNA antibody (ab29. ABCAM) overnight followed by an HRP-conjugated secondary antibody. The mounted slides were observed using a DP-70 microscope digital camera. To analyse BrdU uptake in embryos at E15.5, the mice were injected with BrdU (Sigma) (100 µg/g body weight) and sacrificed 1 hour later. Staining was performed using an anti-BrdU monoclonal antibody (G3G4) at 4°C overnight, followed by an HRP conjugated secondary antibody.

### Phalloidin Staining

The sections were treated with acetone at −20°C for 3 min, washed in PBS three times, and incubated with 1% BSA for 20 min at room temperature, then incubated in rhodamine-phalloidin (6.6 µM) for 5 hours at room temperature, washed in PBS three times and observed immediately using a fluorescence microscope (DP-70, Olympus).

### Real-time Quantitative PCR and Semi-quantitative RT-PCR

The total RNA was isolated from the eyelids of embryos (C57BL/6 and EOB-5 mice) at E14.5, E15.5 and E16.5, respectively. The first-strand cDNA was synthesised by reverse transcriptase M-MLV (Takara) using 1.0 µg of total RNA. Real-time PCR was conducted using the CFX96™ Real-Time PCR Detection System (Bio-rad) and the SYBR Premix Ex Taq (Takara) prepared PCR mixture. The statistical significance between wild-type and EOB-5 samples at each time point was analysed by Student’s t-test. Semi-qPCR was conducted with the Biometra T-Gradient Thermal Cycler. Pax6 and Pax6(5a) were simultaneously amplified with the same sample by using one pair of primers, and a single reference was also amplified.The products of semi-qPCR with the reference were run on an agarose gel. The amount of uploaded product should be the same and within the appropriate volume because either more or less of the product will affect the detection of the CCD. Bands in gels were stained with ethidium bromide and examined under ultraviolet light. A grayscale-scan was performed by GeneTools (SynGene, version 4.01.02) to analyse the relative value compared to the light intensity of the reference. Three biological groups and three technical repeats were performed from PCR amplification to grayscale-scan. The statistical significance of the group was measured using Student’s t-test in the same strain of mice between adjacent time points. All statistical significance was performed using SPSS software, version 17.0.0. The primers used for real-Time PCR were as follows: total Pax6-5′: CTCTATCTTGCGAAAGTTG and total Pax6-3′: CTGAGAACTGGGATATACG; Pax6(5a) -5′: CAGGTGCTGGACAATGAA and Pax6(5a) -3′: CACTCTTGGCTTACTCCCTC; GAPDH-5′: AGGTCGGTGTGAACGGATTTG and GAPDH-3′: TGTAGACCATGTAGTTGAGGTCA. The primers for semi-qPCR were as follows: Pax6e4f: GCTTGGTGGTGTCTTTGTCAAC and Pax6e6r: GATGGAGCCAGTCTCGTAATACC.

## Results

### Identification of the Pax6 Expression Pattern in the Developing Eyelids

**Figure 1:**
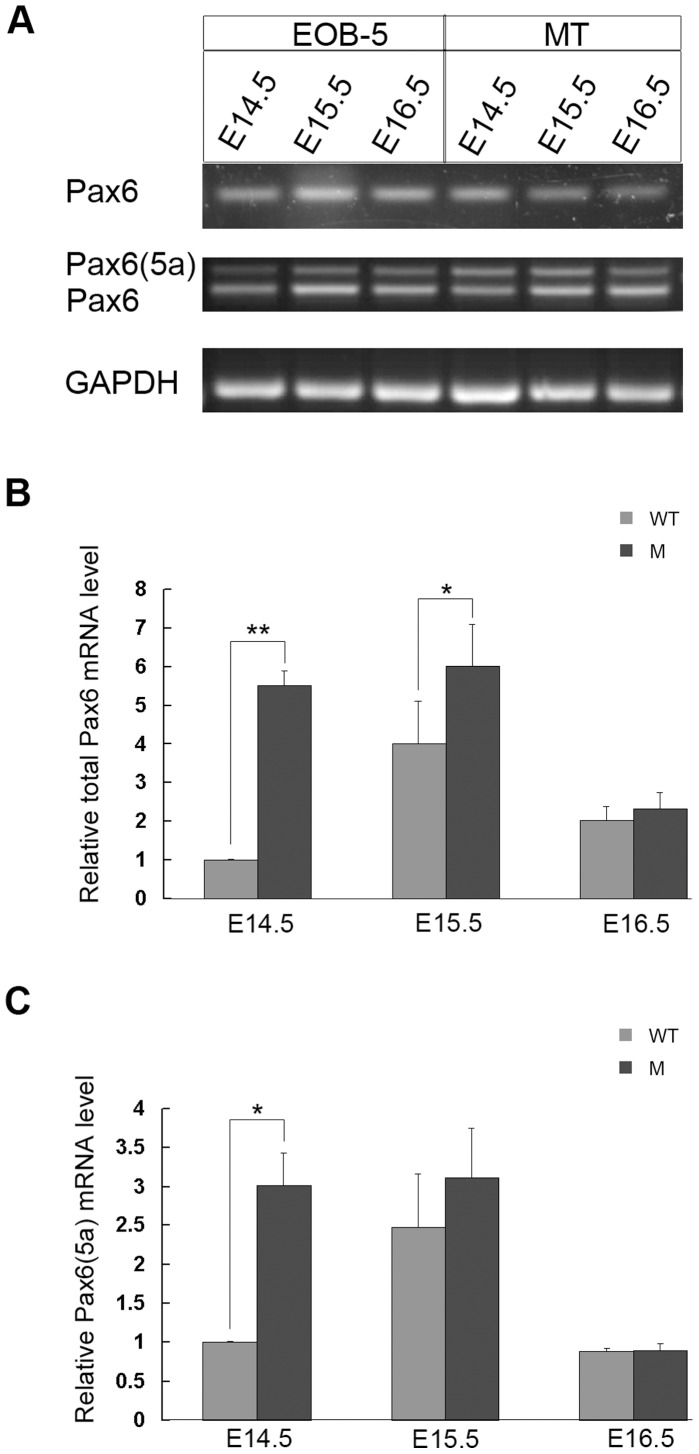
Pax6 mRNA expressed pattern during eyelid development. (A) Semi-quantitative PCR for total expression levels of Pax6 alone and Pax6 and Pax6(5a) using eyelid RNA from E14.5 to E16.5. Pax6 and Pax6(5a) are amplified by the same pair of primers. (B, C) The relative fold expressions of total Pax6 and Pax6(5a) only by real-time PCR, respectively. The reference sample is the cDNA from wild-type eyelids at E14.5. GAPDH is a reference gene. The significantly different data groups at different time point are analysed by Student’s t-test. One asterisk stands for p<0.05, two asterisks mean p<0.01, and no asterisk means no significant difference.

To explore Pax6 expression and its possible expression pattern in the eyelid, mRNA and protein from E14.5 to E16.5 were detected in the wild-type mouse and the EOB-5 mutant mouse, whose eyelid extension was blocked at E15.5. We identified the presence of Pax6 mRNA in eyelids by RT-PCR, which was confirmed by sequencing. The Pax6 expression level in wild-type eyelids was lower than that in EOB-5 eyelids (Fig. 1A). The temporal expression pattern of Pax6 was further verified by real-time PCR during eyelid development at E14.5, E15.5 and E16.5. Pax6 mRNA expression in wild-type eyelids was lowest at E14.5, increased at E15.5, and then declined at E16.5. In comparison, Pax6 mRNA in EOB-5 eyelids was invariably expressed at a high level from E14.5 to E15.5 until there was a sharp reduction at E16.5 (Fig. 1B). The data suggest that the transcriptional change in Pax6 might be related to eyelid development, and relatively low transcription might be required for eyelid development.

**Figure 2:**
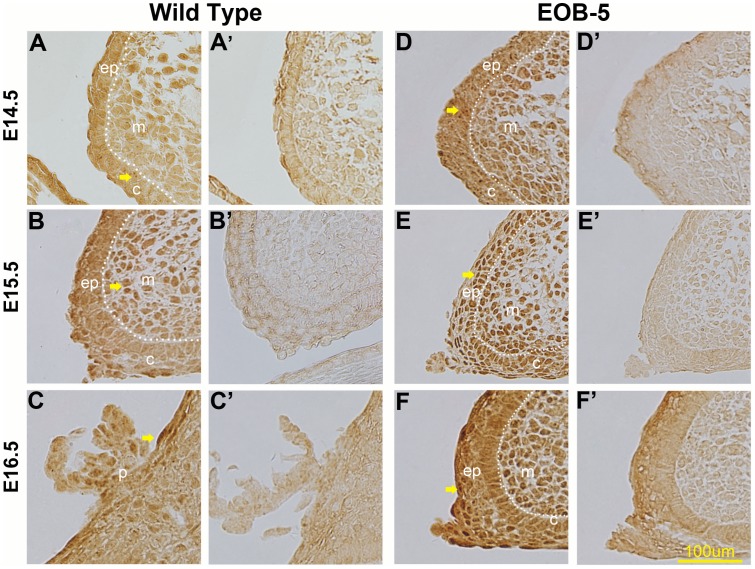
Immunohistochemistry on eyelid sections from E14.5 to E16.5 with anti-PAX6 antibody. (A–C) Pax6 staining in wild-type sections. There is weak but clear staining shown in the conjunctiva and mesenchyme at E14.5 (A); little positive staining is shown in the mesenchymal cells, none is in the epidermis at E15.5 (B), and Pax6 is only in peridermal cells at E16.5. (D–F) Pax6 staining of EOB-5 eyelid sections. There is intense staining in epidermis and mesenchyme at all three days. (A’–F’) Negative controls without primary antibody. Key: ep, epidermis; c, conjunctiva; m, mesenchyme; p, periderm. Bar, 100 µm; arrows indicate positive staining.

To define Pax6 post-transcriptional information and its specific location, we performed immunohistochemistry to examine Pax6 protein expression in the eyelid from E14.5 to E16.5. Significant differences in staining signals and distribution were found between wild-type and EOB-5 eye tissue. In the wild-type eyelid, Pax6 protein was expressed in the nucleus and was detected mainly in the mesenchyme and the conjunctiva with some expression in the epidermis at E14.5 (Fig. 2A). Curiously, Pax6 staining was almost absent in the epithelial layer at E15.5, but there was a small number of positively stained cells in the middle region of the mesenchyme and the tip of the leading edge. Such a significant reduction in Pax6-stained cells contradicts the mRNA level, which was increased at E15.5. At E16.5, the day of eyelid closure, a few Pax6 positive cells re-appeared in the outer surface of the periderm, which was differentiated from epidermis (Fig. 2C). By contrast, in the EOB-5 eyelid, Pax6 nuclear staining was shown in all eyelid tissue, including the epidermis, conjunctiva and mesenchyme, at E14.5 (Fig. 2D), and stronger staining was shown in the epidermal and mesenchymal cells at E15.5 (Fig. 2E). At E16.5, Pax6 staining was restricted to the periderm and some mesenchymal cells of the undeveloped eyelid (Fig. 2F). These results suggest that Pax6 protein, as a transcriptional factor, is negatively correlated with eyelid epidermis development.

### Investigation of Pax6/Pax6 (5a) Ratio and its Correlation with Eyelid Development

The Pax6 gene encodes two isoforms, Pax6 and Pax6(5a). PAX6(5a) is an alternatively spliced product of Pax6 resulting in a 14-amino-acid-residue insertion in the paired domain, which alters the specificity of its DNA-binding activity. The data above actually contain both Pax6 and Pax6(5a). Therefore, we further examined the expression pattern of Pax6(5a) in the developing eyelid from E14.5 to E16.5 by real-time PCR. In wild-type mouse eyelids, the level of Pax6(5a) expression was low at E14.5, increased at E15.5, and decreased at E16.5 (Fig. 1C). By comparison, Pax6(5a) expression in EOB-5 was higher at E14.5 and E15.5 and sharply decreased at E16.5 (Fig. 1C). The data indicate that the expression trend of Pax6(5a) was identical with the tendency of total Pax6 (including Pax6(5a) during normal eyelid development, suggesting that both changed Pax6 and Pax6(5a) mRNA may determine Pax6 gene function.

It has been known that the ratio Pax6/Pax6(5a) is related to the stimulation of some target gene transcription in the eyes and brain [18], [19], [20]. To further explore the possible function of Pax6 differential expression in eyelid development, the two variants of Pax6 were both detected by RT-PCR with one pair of primers, and the Pax6/Pax6 (5a) ratio was calculated by analysing the gel with grayscale-scan. In wild-type mouse eyelids, the ratios were approximately 1.1∶1 at E14.5 and increased from 1.6∶1 at E15.5 to 2.2∶1 at E16.5 (Table 1, Fig. 1A). These increased ratios were statistically significant when each of two age groups was compared (p<0.05). Combined with the result of the relative expression fold of total Pax6 and Pax6(5a) in the wild-type eyelid, this difference indicates that the change in Pax6 mRNA predominantly affects the ratio from E14.5 to E16.5. By comparison, the Pax6/Pax6(5a) ratio was approximately 2∶1 from E14.5 to E16.5 (Table 1, Fig. 1A). Together, these data suggest that the changes in the Pax6/Pax6(5a) ratio may function in the regulation of eyelid initiation, extension and termination (with cell keratinisation) by stimulating their different target genes.

**Table 1:** Distributions of total Pax6 and the ratio of Pax6 and Pax6(5a) in the eyelid.

Mice		E14.5	E15.5	E16.5
WT	Pax6 location	Mesenchyme, Conjunctiva	Mesenchyme, Leading edge	Mesenchyme, Periderm
	Pax6/Pax6(5a)	1.15±0.21	1.61±0.20**	2.19±0.12*
EOB-5	Pax6 location	Mesenchyme, Epidermis, Conjunctiva	Mesenchyme, Epidermis, Leading edge	Mesenchyme, Epidermis, Leading edge
	Pax6/Pax6(5a)	2.62±0.52	2.59±0.46	2.26±0.43

The ratio of Pax6/Pax6(5a) was measured by fluorescence intensity. The significant differences between the values at E14.5 and those at E15.5 and between the values at E15.5 and at E16.5 were analysed in the same sample to illustrate the trend of the ratios during eyelid development (P* = 0.022<0.05, P** = 0.002<0.01).

### Pax6 Expression is Negatively Correlated with the Proliferation of Epidermis Cells

**Figure 3:**
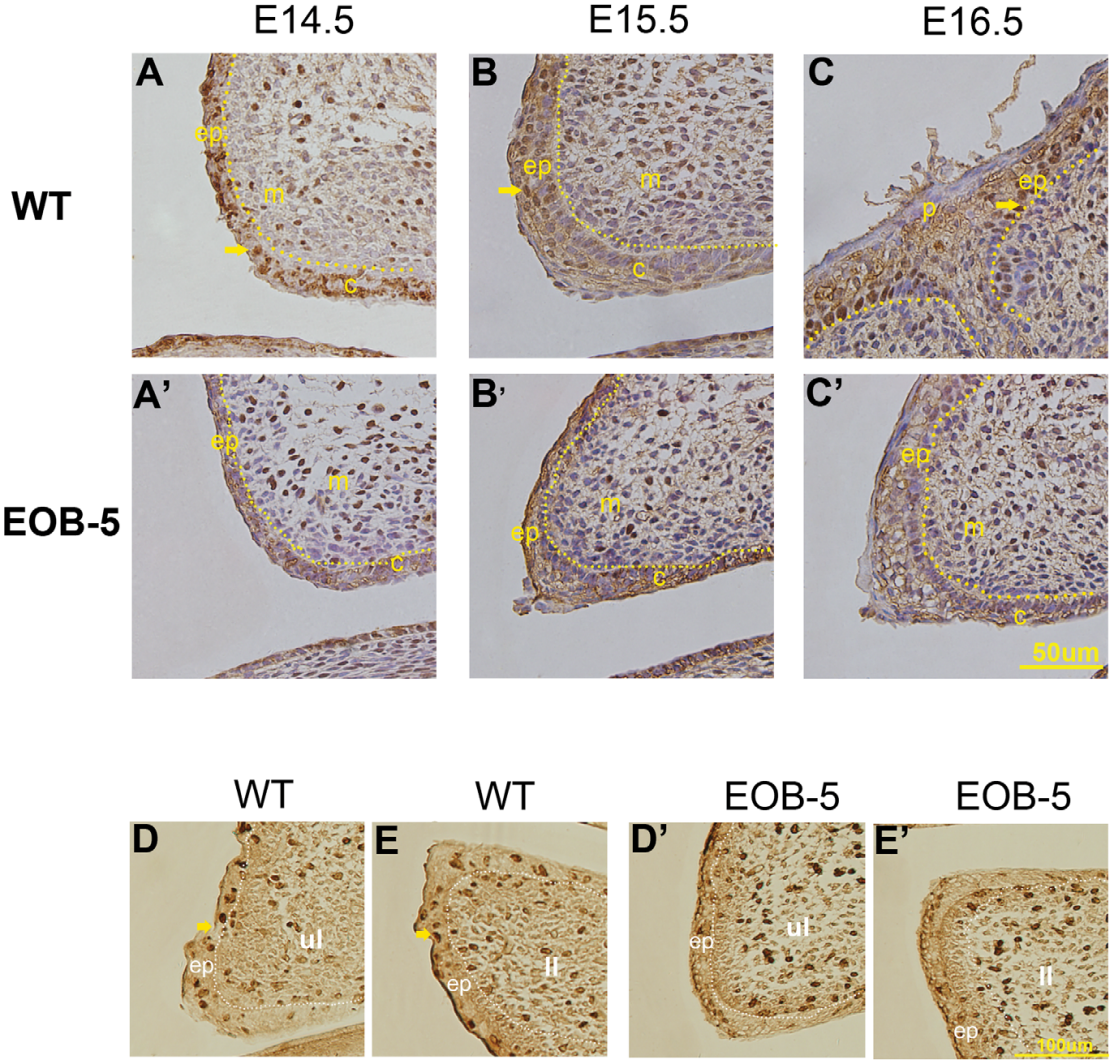
Cell proliferation assays in wild-type and mutant embryos. (A–C and A’–C’) Detection of proliferating cells by PCNA staining. Epidermal cell proliferation presents in wild-type eyelids from E14.5 to E16.5 (A–C) but is almost absent in EOB-5 eyelids (A’–C’). The proliferating epidermal cells were restricted to the basal layer of epidermis in normal eyelid at E16.5 (C). The arrows indicate proliferating cells in the eyelid epidermis. Bar, 50 µm. (D, D’, E, E’) The examination of cell proliferation by Brdu staining in wild-type and mutant embryos at E15.5. Bar, 100 µm. Key: ep, epidermis; ul, upper eyelid; ll, lower eyelid. Bar, 100 µm.

Eyelid development is dependent on cell proliferation, and it has been shown that Pax6 represses corneal epithelial cell proliferation in vitro [21]. To explore whether Pax6 influences cell proliferation in the developing eyelids, the relation between proliferating cells and Pax6 was investigated. Proliferating cells were identified using an anti-PCNA antibody against proliferating cell nuclear antigen (PCNA), a marker of proliferating cells [22]. In normal eyelids, proliferating cells were shown in mesenchyme and epithelium at E14.5 (Fig. 3A), and there were more at E15.5 (Fig. 3B), but the cells were only left in the basal layer of the epidermis at E16.5 (Fig. 3C). Notably, the timing and the location of the proliferating cells in epidermal cells were fully opposite to Pax6 staining. Similarly, in the EOB-5 eyelid from E14.5 to E16.5, proliferating cells were only in the mesenchyme and were absent in the epidermis, where Pax6 was strongly expressed in the nucleus (Fig. 3A’–3C’). To further confirm the result of cell proliferation, E15.5 sections were stained with an anti-BrdU antibody, which is another way to detect proliferating cells [23], and the result was consistent with PCNA staining at E15.5 (Fig. 3D, E). This observation indicates that Pax6 is negatively correlated with epidermal cell proliferation in the developing eyelid.

### Pax6 has no Correlation with Cell Migration in the Eyelid

**Figure 4:**
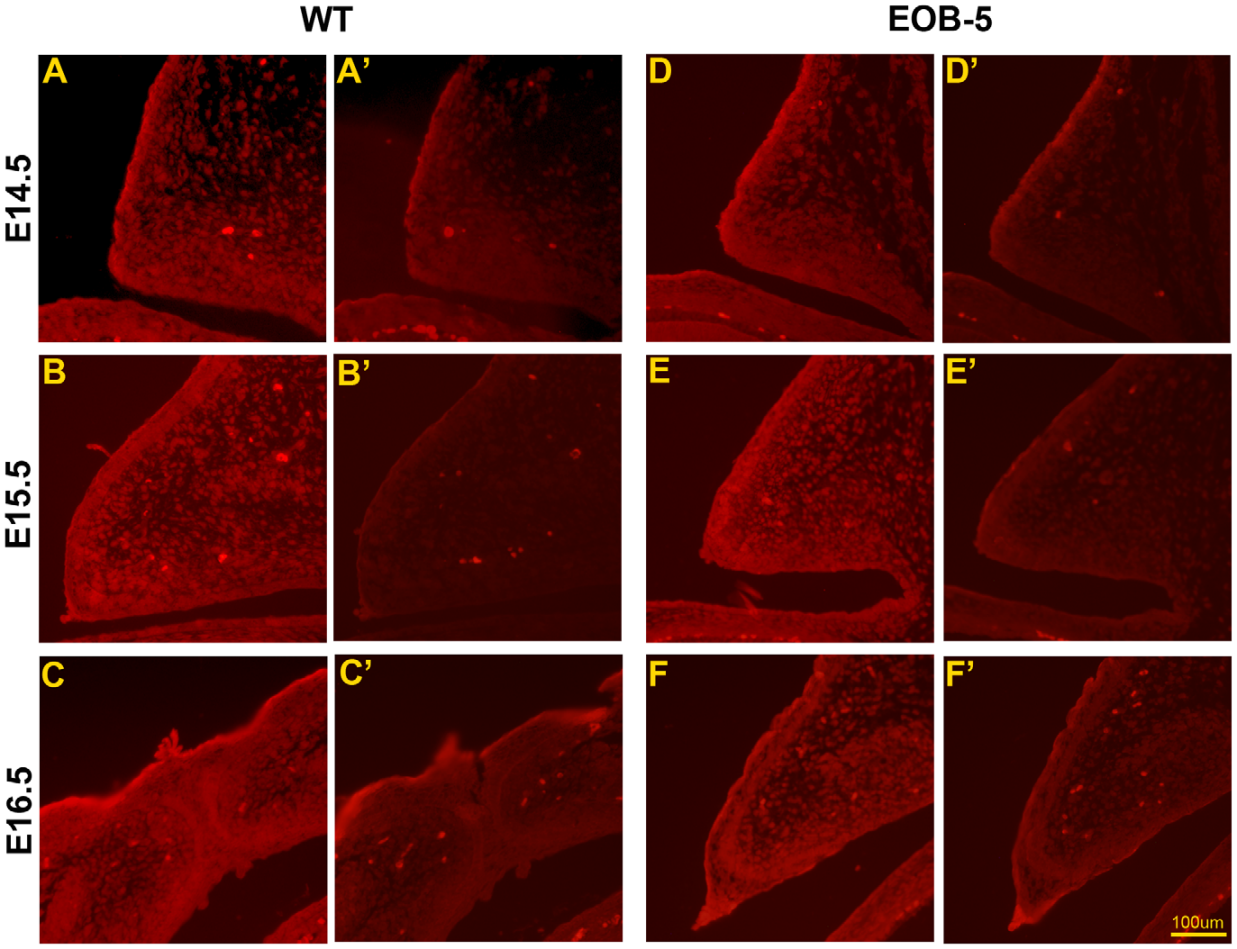
Cell migration in wild-type and mutant eyelids from E14.5 to E16.5. F-actin polymerisation is shown at the margin of the eyelid epithelium in both wild-type (A) and EOB-5 (D) embryos at E14.5. An intense positive staining is shown in the epithelial layer of wild-type (B) and EOB-5 (E) eyelids at E15.5. F-actin polymerisation was reduced when eyelid fusion was completed. More intense staining of polymerised F-actin was detected in the wild-type (C) embryo at E16.5 and in the un-fused eyelid of EOB-5 (F) embryo at E16.5. (A’–F’) Negative controls without phalloidin. Rhodamine-phalloidin was used to visualise F-actin (red). Bar, 100 µm.

In addition to cell proliferation, eyelid development also requires cell migration, which can be identified through the detection of actin polymerisation with phalloidin staining [24], [25]. To determine whether Pax6 is correlated with cell migration in the developing eyelid, we assessed the location of polymerised F-actin from E14.5 to E16.5 and compared its location with the results of Pax6 staining. In the wild-type eyelid, F-actin polymerisation appeared at the margin of the eyelid epithelium E14.5 (Fig. 4A), grew more intense in the epidermal cells at E15.5 (Fig. 4B) and E16.5 when the eyelid fused (Fig. 4C). The detection of polymerised F-actin in the EOB-5 eyelid at E14.5, E15.5 and E16.5 (Fig. 4A’, B’, C’) was identical to the wild-type. Compared with results of Pax6 staining, it showed that neither positive nor negative correlation has existed between Pax6 and cell migration.

### Pax6 is Negatively Correlated with the Activity of the EGFR-ERK Pathway

**Figure 5:**
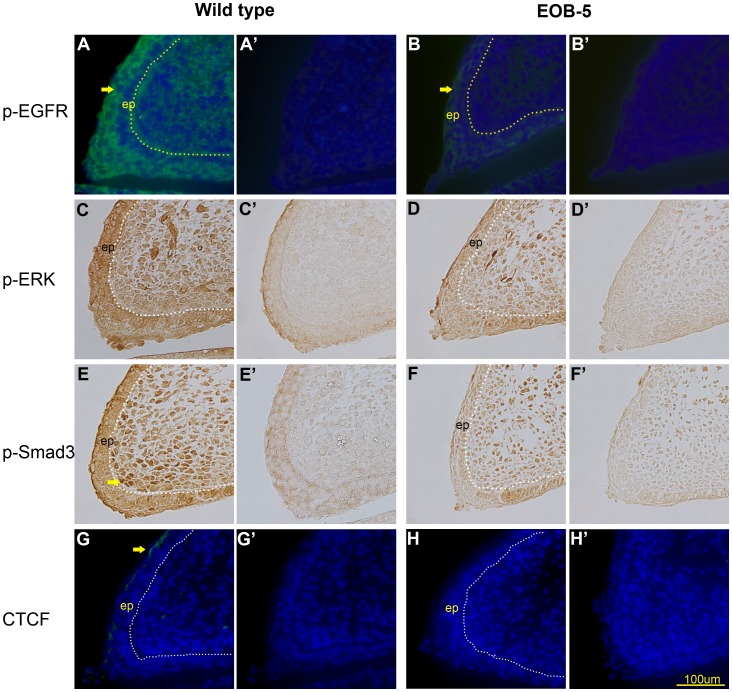
The examination of the EGFR-ERK pathway and Smad3 activation in eyelid epidermis at E15.5. (A–D) The activation state of EGFR and its downstream signalling molecules in the epidermis of wild-type and EOB-5 eyelids. Phosphorylated EGFR and ERK were predominantly stained in wild-type epidermis (A, C), while this staining was weak in the mutant eyelid (B, D). (E, F) The activation state of Smad3. Phosphorylated Smad3 was strongly expressed in the mesenchyme underlying the epidermis of wild-type eyelids (E) and was weakly expressed in the mesenchyme of EOB-5 eyelids (F). (G, H) The examination of CTCF expression. A positive signal (green) was shown in the epidermis of the wild-type eyelid (G), while no positive staining was shown in the mutant eyelid (H). (A’–H’) Negative controls (no primary antibody). Key: ep, epidermis; c, conjunctiva; m, mesenchyme; p, periderm. Bar, 100 µm; arrows indicate positive staining.

Pax6 expression in mouse corneal epithelial cells is thought to be inhibited by the EGFR-ERK signalling pathway, which is involved in eyelid migration and proliferation according to studies using EOB mice [15], [26]. Therefore, to assess whether the EGFR-ERK pathway affects Pax6 expression in eyelids, we examined activated EGFR and ERK (with phosphorylation) in wild-type and EOB-5 eyelids at E15.5 because the dramatic difference in Pax6 expression between the two tissue types was shown at that day. In wild-type eyelid, strong staining of phosphorylated EGF receptor (pEGFR) and p-ERK (Fig. 5A, C) were shown in the epidermis where Pax6 protein was absent, whereas, in the EOB-5 epidermis, significant reductions in pEGFR and pERK (Fig. 5B, D) were accompanied by high Pax6 expression. The results indicate a negative correlation between Pax6 and the EGFR-ERK pathway. To enhance this notion, we further investigate the co-localisation between Pax6 and CTCF, an EGF-induced transcription factor involved in the repression of Pax6 in corneal cells [26], [27]. As expected, in the wild-type eyelid, CTCF was mainly detected in the nuclei of epidermal cells where Pax6 was not expressed (Fig. 5G), while it was barely was detected in EOB-5 epidermis with prominent nuclear expression of Pax6 (Fig. 5H). These results suggest that the EGFR-ERK pathway might negatively regulate Pax6 expression in the epidermis.

Although it is known that Pax6 transcription is regulated by two promoters, P0 and P1, which are the functional control regions of spatial and temporal expression of Pax6 in development [28] and are regulated by a long range downstream enhancer [29] and autoregulation [30], little is known about its targeting regulatory protein. Smad3, a receptor-associated Smad family protein, is suggested to regulate the ovarian surface epithelium morphological appearance and proliferation [31] and can bind to the paired domain of Pax6 to repress the autoregulation of the Pax6 P1 promoter in FHL124 cells [30]. We therefore investigated the possible relationship between Pax6 and activated Smad3 (phosphorylated Smad3, p-Smad3) in the eyelid at E15.5. As shown in Fig. 5E, phosphorylated Smad3 was remarkably expressed in the mesenchymal cells of the normal eyelid close to the epithelial layer, while it was absent in the EOB-5 eyelid (Fig. 5F). Such a distribution of expression was not correlated with Pax6 expression, indicating that Pax6 could not be regulated by Smad3 in the eyelid, although the distinct expression between the EOB-5 and the normal eyelid showed the involvement of pSmad3 in eyelid formation.

## Discussion

Eyelid morphogenesis is induced by the cornea after visual system formation. Although Pax6 is expressed in the cornea and other eye tissues to control eye morphogenesis [3], [21], previous data have not defined whether Pax6 continues to play a role in eyelid induction and development after cornea formation. In this study, we confirm that Pax6 coupled with Pax6(5a) is still expressed in the eyelid. Comparison research on the normal and EOB-5 mutant mouse has shown that Pax6 expression is closely correlated with epidermal cells proliferation and with the activation of EGFR-ERK pathways. Moreover, relatively low expression and a low ratio of Pax6/Pax6(5a) has been shown in the normally developing eyelid.

A clear negative correlation between Pax6 protein expression and epidermal cell proliferation is shown in Fig. 2B, 2E and Fig. 3D, 3D’. Increased expression of the Pax6 protein in the EOB-5 eyelid significantly inhibits epidermal cell proliferation, which is consistent with previous studies showing that Pax6 overexpression inhibits cell proliferation in murine corneal [9], iris, and ciliary body cells [10] and astrocytes [32], as well as an in vitro study showing that Pax6 overexpression inhibits the growth of rabbit corneal epithelial cells [33]. We postulate that a moderately low level of Pax6 protein is required for eyelid development. Although Pax6 expression is negatively correlated with the proliferation of epidermal cells, it appears that it is not correlated with that of mesenchymal cells, suggesting that the manner of Pax6 function might be related to different differentiated cell lineages. In the normal eyelid, by analysing the location of the Pax6 protein, positive nuclear staining at E14.5 is mainly presented in mesenchyme and conjunctiva cells, but it is absent at E15.5 (Fig. 2A, B), which means that Pax6, as a transcriptional factor, may function in the beginning of eyelid formation. When the eyelids are fully closed at E16.5, Pax6 is specifically expressed in the keratinocyte cells of the eyelid periderm (Fig. 2C), indicating that it may function in cellular differentiation. These data together suggest that the Pax6 protein only functions during the initiation and termination of eyelid development and does not contribute to the developing eyelid at E15.5. It is necessary to note that the Pax6 mRNA level disagrees with the Pax6 protein in the normal eyelid at E15.5. This discrepancy might be due to post-transcriptional or translational regulation, as in a recent report showing that miR-7a can restrict Pax6 protein expression in neural stem cells [34]. In contrast to the wild-type mouse, the Pax6 protein in EOB-5 eyelids is correlated with its mRNA level, implying that the posttranslational control is blocked in EOB-5 mice. Because there is no commercial antibody specific for that individual isoform available, we were unable to distinguish Pax6 from Pax6(5a) at the protein level in this research, and the PAX6 differential expression pattern and function in the eyelid needs to be further studied. Despite this limitation, the anomalously high expression of Pax6 in the EOB-5 eyelid suggests that the relatively low Pax6 expression in the normal eyelid is required for cell proliferation and that the up-regulation of Pax6 by disruption of expression regulation can cause an inhibition of epidermal proliferation leading to an eyes-open-at birth (EOB) phenotype.

Differential gene expression with different isoforms is important for developmental regulation. The limited available data suggest that the balance of Pax6/Pax6(5a) is essential for normal lens physiology in vivo [35]. Disrupting this balance by the overexpression of human PAX6(5a) or mutation in the 5a exon causes an anomalous lens, which can be observed in congenital cataracts [36], [37]. During normal murine neurogenesis, Pax6/Pax6(5a) displays a decreasing expression trend from 6–10 times to approximately 3∶1. In eye development, the ratio varies from approximately 10∶1 at E14.5 to approximately 4∶1 at E18.5, which are considered to be insignificant changes [35]. In our study, the Pax6/Pax6(5a) ratio in the eyelid is lower, gradually changed from 1.15∶1 at E14.5, 1.61∶1 at E15.5 and 2.19∶1 at E16.5. Therefore, relatively low expression of Pax6 and the Pax6/Pax6(5a) ratio may reflect the Pax6 function in eyelid development. Noticeably, at a time when the normal eyelid completes closure, the Pax6/Pax6(5a) ratio of 2∶1 is similar to that in the EOB-5 eyelid, in which development is blocked due to the inhibition of epidermis cell proliferation. These data imply that the susceptible regulation of Pax6/Pax6 (5a) from 1.61∶1 up to 2.19∶1 is competent to inhibit, or at least to help inhibit, eyelid epidermis cell proliferation and improve cell differentiation. Therefore, the Pax6/Pax6(5a) ratio may represent a balance in eyelid development. Once the balance is disrupted, the gene expression pattern regulated by these two transcriptional factors will lead to another direction of cell development. However, such a ratio change might simply illustrate a relationship with the functional Pax6 protein in developing eyelid because mRNA levels may not easily match the protein levels and may not reflect the change in protein distribution. Hence, more detailed research is required to understand the complexity of Pax6 function in eyelid development.

Although many differentially expressed isoforms have been found and are able to regulate the transcription or protein activation of the ‘wild-type’ isoform [38], it is thought that there is not antagonistic action between Pax6 and Pax6(5a) because they have their own set of target genes during development [39]. Moreover, PAX6(5a) is unable to bind to the sites for Pax6, and Pax6 binds to the sites for PAX6(5a) as a monomer displaying less efficiency [40]. However, a functional interaction between Pax6 and Pax6(5a) has been found to synergistically enhance the activation of the crystalline promoter in mouse [41]. Moreover, in the 3T3 cell line, which stably expresses Pax6 or Pax6(5a), Pax6-upregulated genes are associated with cell growth and adhesion, while Pax6(5a)-upregulated genes are correlated with cell motion and migration [39]. In this study, Pax6 and Pax6(5a) display a synergistic expression pattern. Taken together, it appears that the Pax6 gene encodes Pax6 and Pax6(5a), which are independent in regulating their respective targeting genes, and subsequently, the activation of these downstream genes contributes to coordination in eyelid development. The remaining question is to determine the detailed underlying mechanism for the regulation of the Pax6/Pax6(5a) expression ratio.

It is clear that the EGFR-ERK pathway contribute to eyelid development [15]. The correlations between Pax6 and the EGFR-ERK pathway, between Pax6 and cell proliferation, and between the EGFR-ERK pathway and cell proliferation are clearly shown in this study. The reduction in EGFR and ERK activity, with a low level of CTCF expression in the epidermis of EOB-5 mice, is accompanied by abnormally high expression of Pax6. Combined with previous in vitro reports that show that down-regulation of Pax6 in corneal epithelial cells is required for EGF-induced cell proliferation [25] and that EGF stimulates ERK activity to increase CTCF expression to inhibit Pax6 in corneal epithelial cells [26], we speculate that relatively low Pax6 expression is required for eyelid development and that this expression is controlled by the EGFR-ERK pathway. Together with evidence that Pax6 is negatively correlated with cell proliferation but not cell migration (Fig 3, 4), this observation shows that the EGFR-ERK-Pax6 pathway is important for the regulation of epidermal cell proliferation. As the EGFR-ERK pathway has been known also to regulate epithelial cell migration in the eyelid [16], it is likely that Pax6 is regulated by one cascade of the EGFR-ERK pathway in the eyelid.

In summary, our present data suggest that the initiation of mouse eyelid formation requires relatively low expression levels of Pax6 at E14.5 and that cell proliferation at E15.5 requires an inhibition of Pax6 expression. A comparatively low ratio of Pax6/Pax6(5a) may represent the function of Pax6 in eyelid development. The EGFR-ERK pathway is involved in the negative regulation of Pax6. These data offer insight into Pax6 function and its mechanism of regulation in the developing eyelid.
